# Complete Genome Sequences of 11 *Haemophilus ducreyi* Isolates from Children with Cutaneous Lesions in Vanuatu and Ghana

**DOI:** 10.1128/genomeA.00459-16

**Published:** 2016-07-07

**Authors:** Allan Pillay, Samantha S. Katz, A. Jeanine Abrams, Ronald C. Ballard, Shirley V. Simpson, Fasihah Taleo, Monica M. Lahra, Dhwani Batra, Lori Rowe, David L. Trees, Kingsley Asiedu, Cheng-Yen Chen

**Affiliations:** a Laboratory Reference and Research Branch, Division of Sexually Transmitted Diseases Prevention, NCHHSTP, Centers for Disease Control and Prevention, Atlanta, Georgia, USA; b Center for Global Health, Centers for Disease Control and Prevention, Atlanta, Georgia, USA; c Noguchi Memorial Institute for Medical Research, University of Ghana, Legon, Ghana; d Ministry of Health, Port Vila, Vanuatu; e World Health Organization Collaborating Centre for STD, Microbiology Department, The Prince of Wales Hospital, Sydney, Australia; f Biotechnology Core Facility Branch, Division of Scientific Resources, NCEZID, Centers for Disease Control and Prevention, Atlanta, Georgia, USA; g Department of Control of Neglected Tropical Diseases, World Health Organization, Geneva, Switzerland

## Abstract

*Haemophilus ducreyi* causes chancroid and has recently been shown to be a significant cause of cutaneous lesions in tropical or subtropical regions where yaws is endemic. Here, we report the draft genome assemblies for 11 cutaneous strains of *Haemophilus ducreyi*, isolated from children in Vanuatu and Ghana.

## GENOME ANNOUNCEMENT

*Haemophilus ducreyi* is a fastidious Gram-negative bacterium that causes chancroid, a sexually transmitted disease characterized by painful genital ulcers. The global prevalence of chancroid has declined significantly in the past decade due to syndromic management of genital ulcer disease (1). There have been sporadic reports of cutaneous lesions due to nonsexual transmission of *H. ducreyi* (2, 3), but recent surveys, as part of the WHO yaws eradication program, have shown a high prevalence in the South Pacific islands and Ghana (4–6).

Very little is known about *H. ducreyi* strains responsible for cutaneous lesions in children. To better understand the genetic differences between genital and cutaneous strains of *H. ducreyi* from different geographic locations, we performed whole-genome sequencing on cutaneous strains isolated in 2014 and 2015 from children in Vanuatu and Ghana during yaws surveys.

Lesion swabs were streaked onto Columbia agar plates containing 1% hemoglobin (BBL, Franklin Lakes, NJ, USA), 0.2% activated charcoal (Sigma-Aldrich, St. Louis, MO, USA), 5% fetal bovine serum (Atlanta Biologicals, Atlanta, GA, USA), and 1% IsoVitaleX (BBL), and incubated in a sealed paint can (candle jar) under CO_2_ conditions. Plates were transferred to the laboratory and incubated for 48 h at 33°C under microaerophilic conditions. In Vanuatu, all bacterial colonies were scraped off primary plates, transferred to a transport medium (7), and transported on ice packs to the WHO Collaborating Centre for STD, Sydney. *H. ducreyi* was isolated on Columbia agar plates and identified by 16S rRNA sequencing. In Ghana, bacteria from primary plates or suspected *H. ducreyi* colonies were frozen in storage medium containing 1% proteose peptone no. 3 (BD, Franklin Lakes, NJ, USA) and 0.8% glycerol and shipped to the CDC for identification using biochemical tests and PCR (8).

DNA was extracted using the ArchivePure DNA cell/tissue kit (5 PRIME, Inc., Gaithersburg, MD, USA) following the manufacturer’s guidelines. Whole-genome sequencing was conducted using the PacBio RSII platform (Pacific Biosciences, Menlo Park, CA, USA) with P6-C4 and P6 v2-C4 chemistry. A single-molecule real-time (SMRT) cell was used to sequence each genome, and *de novo* assembly of the genomes was conducted using the hierarchical genome assembly process (HGAP3, SMRTAnalysis version 2.3.0) workflow, which included consensus-polishing using Quiver (9). Sequences were annotated using the NCBI Prokaryotic Genome Annotation Pipeline (PGAP version 3.1, http://www.ncbi.nlm.nih.gov/genome/annotation_prok). Mean coverage, assembly size, G+C content, numbers of contigs and predicted coding sequences and RNAs, as well as accession numbers can be found in Table 1. A comparative analysis of these genomes will be described in a future publication.

**TABLE 1:** Summary characteristics of whole-genome assemblies

Strain^a^	Mean coverage (×)	No. of contigs	Assembly size (bp)	G+C content (%)	No. of coding sequences and RNAs	Accession no.
VAN1	78	1	1,667,451	38.1	1,634	CP015424
VAN2	79	1	1,589,620	37.9	1,536	CP015425
VAN3	71	2	1,667,096	38.1	1,629	CP015426
VAN4	74	2	1,673,048	38.1	1,642	CP015427
VAN5	77	2	1,667,484	38.1	1,635	CP015428
GHA1	74	1	1,622,156	37.9	1,552	CP015429
GHA2	187	1	1,634,243	37.9	1,561	CP015430
GHA3	132	3	1,738,543	38.2	1,709	CP015431
GHA5	257	1	1,738,559	38.2	1,717	CP015432
GHA8	58	1	1,769,925	38.2	1,745	CP015433
GHA9	223	1	1,775,503	38.2	1,753	CP015434

^a^ VAN, Vanuatu; GHA, Ghana.

### Nucleotide sequence accession numbers.

The complete genome sequences for the five Vanuatu and six Ghana cutaneous *H. ducreyi* strains have been deposited in GenBank under the accession numbers listed in Table 1.
